# Supplementary material for: Exploring Feedback Mechanics during Experiential Learning in Pharmacy Education: A Scoping Review
Source: Pharmacy (Basel). 2024 May 7;12(3):74. doi: 10.3390/pharmacy12030074 (PMC11130841; doi:10.3390/pharmacy12030074)
Supplement: Supplementary file 1 [file pharmacy-12-00074-s001.zip › pharmacy-2916173-supplementary.pdf]

pharmacy MDPI

Supplementary Materials

Table S1. Search strategy for PubMed

Search number	Query	Results
4	(((Pharmacy student OR PharmD student OR pharmacy resident OR pharmacy fellow OR pharmacy trainee OR Bpharm OR MPharm) AND (Feedback OR Formative feedback)) AND (Experiential education OR experiential teaching OR clinical rotation OR internship OR residency OR fellowship OR bedside teaching OR clerkship OR clinical clerkship OR workplace based learning)	387
3	Experiential education OR experiential teaching OR clinical rotation OR internship OR residency OR fellowship OR bedside teaching OR clerkship OR clinical clerkship OR workplace-based learning	535,735
2	Feedback OR Formative feedback	194,420
1	Pharmacy student OR PharmD student OR pharmacy resident OR pharmacy fellow OR pharmacy trainee OR Bpharm OR MPharm	28,807

Table S2. Search strategy for Web of Science.

Search number	Query	Results
4	(#1) AND (#2) AND (#3)	248
3	Experiential education OR experiential teaching OR clinical rotation OR internship OR residency OR fellowship OR bedside teaching OR clerkship OR clinical clerkship OR workplace-based learning (All Fields)	1,231,159
2	Feedback OR Formative feedback (All Fields)	642,719
1	Pharmacy student OR PharmD student OR pharmacy resident OR pharmacy fellow OR pharmacy trainee OR Bpharm OR MPharm (All Fields)	30781

Table S3. Search strategy for Embase.

Search number	Query	Results
3	('pharmacy student'/exp OR 'pharmacy student' OR (('pharmacy'/exp OR pharmacy) AND ('student'/exp OR student)) OR 'pharmd student' OR (pharmd AND ('student'/exp OR student)) OR (('pharmacy'/exp OR pharmacy) AND resident*) OR (('pharmacy'/exp OR pharmacy) AND fellow*) OR (('pharmacy'/exp OR pharmacy) AND trainee*) OR bpharm OR mpharm) AND (feedback OR (formative AND feedback)) AND (experiential AND education OR (experiential AND teaching) OR (clinical AND rotation) OR internship OR residency OR fellowship* OR (bedside AND teaching) OR clerkship OR (clinical AND clerkship) OR (workplace AND based AND learning))	438
2	('pharmacy student'/exp OR 'pharmacy student' OR (('pharmacy'/exp OR pharmacy) AND ('student'/exp OR student)) OR 'pharmd student' OR (pharmd AND ('student'/exp OR student)) OR (('pharmacy'/exp OR pharmacy) AND resident*) OR (('pharmacy'/exp OR pharmacy) AND fellow*) OR (('pharmacy'/exp OR pharmacy) AND trainee*) OR bpharm OR mpharm) AND (feedback OR (formative AND feedback))	1,952
1	'pharmacy student'/exp OR 'pharmacy student' OR (('pharmacy'/exp OR pharmacy) AND ('student'/exp OR student)) OR 'pharmd student' OR (pharmd AND ('student'/exp OR student)) OR (('pharmacy'/exp OR pharmacy) AND resident*) OR (('pharmacy'/exp OR pharmacy) AND fellow*) OR (('pharmacy'/exp OR pharmacy) AND trainee*) OR bpharm OR mpharm	38,540

Table S4. Search strategy for Academic Search Ultimate (EBSCO).

Search number	Query	Results
4	(Experiential education OR experiential teaching OR clinical rotation OR internship OR residency OR fellowship OR bedside teaching OR clerkship OR clinical clerkship OR workplace based learning) AND (Feedback OR Formative feedback) AND (Pharmacy student OR PharmD student OR pharmacy resident OR pharmacy fellow OR pharmacy trainee OR Bpharm OR MPharm)	69
3	Experiential education OR experiential teaching OR clinical rotation OR internship OR residency OR fellowship OR bedside teaching OR clerkship OR clinical clerkship OR workplace-based learning	114,764
2	Feedback OR Formative feedback	215,530
1	Pharmacy student OR PharmD student OR pharmacy resident OR pharmacy fellow OR pharmacy trainee OR Bpharm OR MPharm	6099

Table S5. Search strategy for EBSCO (ERIC).

Search number	Query	Results
3	(pharmacy student OR pharmd student OR pharmd OR resident OR fellow OR trainee OR bpharm OR mpharm) AND (feedback OR formative feedback) AND (experiential education OR experiential teaching OR clinical rotation OR internship OR residency OR fellowship OR bedside teaching OR clerkship OR clinical clerkship OR workplace based learning)	130
2	(pharmacy student OR pharmd student OR pharmd OR resident OR fellow OR trainee OR bpharm OR mpharm) AND (feedback OR formative feedback)	983
1	pharmacy student OR pharmd student OR pharmd OR resident OR fellow OR trainee OR bpharm OR mpharm	26,172

Table S6. Search strategy for ProQuest Central.

Search number	Query	Results
4	(Experiential education OR experiential teaching OR clinical rotation OR internship OR residency OR fellowship OR bedside teaching OR clerkship OR clinical clerkship OR workplace based learning) AND (Feedback OR Formative feedback) AND (Pharmacy student OR PharmD student OR pharmacy resident OR pharmacy fellow OR pharmacy trainee OR Bpharm OR MPharm) =abstract only	71
3	Experiential education OR experiential teaching OR clinical rotation OR internship OR residency OR fellowship OR bedside teaching OR clerkship OR clinical clerkship OR workplace-based learning	1061764
2	Feedback OR Formative feedback	1354307
1	Pharmacy student OR PharmD student OR pharmacy resident OR pharmacy fellow OR pharmacy trainee OR Bpharm OR MPharm	137188
